# Non-injection resection using bipolar soft coagulation mode for large colorectal polyps including incidental cancer

**DOI:** 10.1055/a-2679-6546

**Published:** 2025-09-04

**Authors:** Mitsuo Tokuhara, Yasushi Sano, Yoshifumi Watanabe, Ikuko Torii, Katsuyasu Kouda, Makoto Naganuma

**Affiliations:** 1 13631 Department of Gastroenterology, JCHO Hoshigaoka Medical Center, Hirakata, Japan; 2 157511 Third Department of Internal Medicine, Division of Gastroenterology and Hepatology, Kansai Medical University Hirakata Hospital, Hirakata, Japan; 3 Gastrointestinal Center, Sano Hospital, Kobe, Japan; 4 13631 Department of Pathology, JCHO Hoshigaoka Medical Center, Hirakata, Japan; 5 12880 Department of Hygiene and Public Health, Kansai Medical University, Hirakata, Japan

Large colorectal polyps require complete resection because of their high cancerous potential 1 2 3 , but the current methods of endoscopic snare resection are time-consuming and carry a high risk of incomplete resection and adverse events 4 5 . This study evaluated non-injection resection using bipolar soft coagulation mode (NIRBS) for large colorectal polyps (≥10 mm) in terms of its ability to achieve complete resection, safety, and simplicity.

This study included large colorectal polyps (≥10 mm) that were resected by NIRBS ( Fig. 1 , Video 1 ) from September 2021 to January 2024. Exclusion criteria for resection were cancerous polyps judged to be infiltrating deeper than the submucosal layer endoscopically and laterally spreading-type polyps larger than 20 mm. The absolute value of the diameter and shape of polyps except for laterally spreading tumors (LSTs) were irrelevant. Antithrombotic drugs were temporarily suspended before the procedure.

The video shows non-injection resection using bipolar soft coagulation mode for large colorectal polyps (≥10 mm).Video 1

**Fig. 1:**
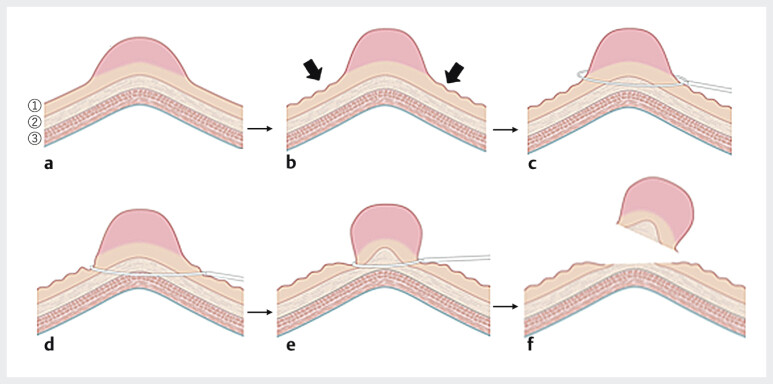
Non-injection resection using bipolar soft coagulation mode (NIRBS) procedure 1. **a** A polyp. 1 Mucosal layer. 2 Submucosal layer. 3 Muscle layer. **b** Direction of the pressure. There is less mucosal pressure and tension since submucosal injection is omitted and air in the lumen is suctioned. An angle is formed at the boundary between the lesion and the normal mucosa. **c** Snaring. **d** The snare grips the mucosa without slipping because of the decreased mucosal pressure and tension. **e** The snare squeezes the submucosal layer, sliding just above the muscle layer without involving it due to the rapid juggling, and then the snare is energized by squeezing. **f** The polyp is resected without residual lesions.

NIRBSs were performed for 185 polyps (mean diameter 13.5 ± 4.5 mm). There were three cases of delayed bleeding (1.6%, 3/185), but no perforation occurred. The mean procedure time was about one minute. The en bloc and R0 resection rates were 95.1% (176/185) and 94.1% (174/185), respectively. En bloc and R0 resection were achieved in all 13 incidentally cancerous lesions (mean size: 18.8 mm) with sufficient margin for the reasons in Fig. 2 .

**Fig. 2:**
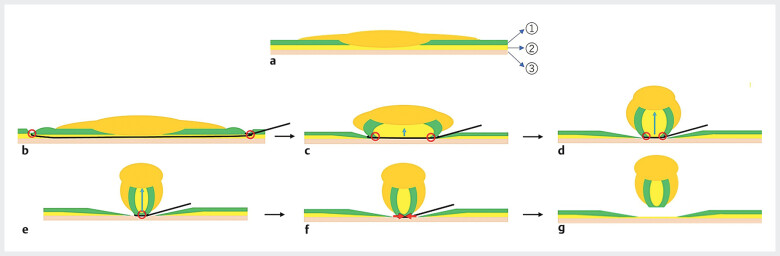
NIRBS procedure 2. **a** A flat elevated lesion. 1 Mucosal layer. 2 Submucosal layer (softest in the three layers). 3 Muscle layer. **b** Snaring. **c–e** The snare squeezes the polyp, sliding just above the muscle layer. The softest submucosal layer is lifted upward by the same force that strangles the mucosal layer toward the center of the snare (↑). The strangled mucosal layers eventually adhere to each other (○), completely separating the deepest portion of the polyp from the muscular layer. Unless the tumor has invaded the muscle layer, it is unlikely to have positive vertical margins. **f** Energization by squeezing. **g** Resected tumor.

The same resection method as NIRBS should not be performed with any settings other than soft coagulation mode, even if a bipolar snare is used, because there is a risk of perforation if the Forced mode or Endocut mode is used in this technique ( Fig. 3 ).

**Fig. 3:**
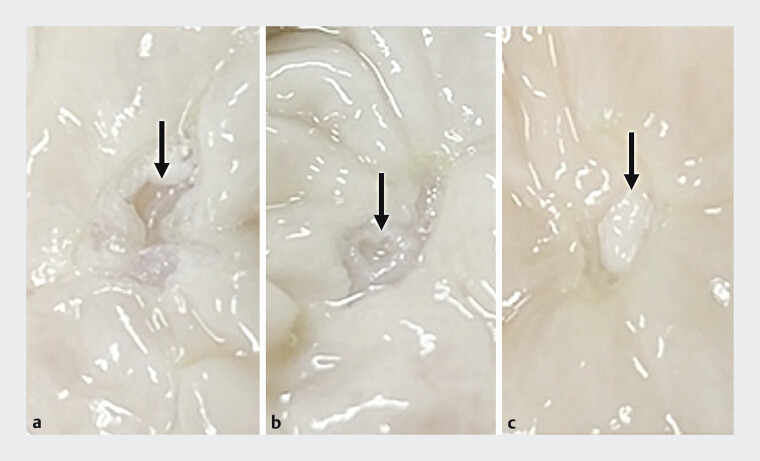
Comparison of each electrosurgery generator mode using a bipolar snare in the porcine colon. **a** Forced (Effect 4.5). The muscle layer is perforated. **b** Endocut Q (Effect 3, Duration 1, Interval 6). The muscle layer is damaged. **c** NIRBS. The submucosal layer just above the muscle layer was completely resected without damaging the muscle layer.

NIRBS is an effective, safe, and rapid method that can achieve the complete resection of large and various forms of colorectal polyps, even those with cancerous potential.

Endoscopy_UCTN_Code_TTT_1AQ_2AD_3AB

## Notes

Endoscopy E-Videos https://eref.thieme.de/e-videos
*E-Videos* is an open access online section of the journal *Endoscopy* , reporting on interesting cases and new techniques in gastroenterological endoscopy. All papers include a high-quality video and are published with a Creative Commons CC-BY license. Endoscopy E-Videos qualify for HINARI discounts and waivers and eligibility is automatically checked during the submission process. We grant 100% waivers to articles whose corresponding authors are based in Group A countries and 50% waivers to those who are based in Group B countries as classified by Research4Life (see: https://www.research4life.org/access/eligibility/ ). This section has its own submission website at https://mc.manuscriptcentral.com/e-videos .
